# Tricuspid Regurgitation in the Era of Transcatheter Interventions: The Pivotal Role of Multimodality Imaging

**DOI:** 10.3390/jcm14145011

**Published:** 2025-07-15

**Authors:** Valeria Maria De Luca, Stefano Censi, Rita Conti, Roberto Nerla, Sara Bombace, Tobias Friedrich Ruf, Ralph Stephan von Bardeleben, Philipp Lurz, Fausto Castriota, Angelo Squeri

**Affiliations:** 1 GVM Care & Research, Maria Cecilia Hospital, 48033 Cotignola, Italy; scensi@gvmnet.it (S.C.); rconti@gvmnet.it (R.C.); rnerla@gvmnet.it (R.N.); fcastriota@gvmnet.it (F.C.); asqueri@gvmnet.it (A.S.); 2 Department of Cardiology, University Medical Center Mainz, 55131 Mainz, Germany; sara.bombace@unimedizin-mainz.de (S.B.); tobias.ruf@unimedizin-mainz.de (T.F.R.); stephan.von_bardeleben@unimedizin-mainz.de (R.S.v.B.); philipp.lurz@unimedizin-mainz.de (P.L.)

**Keywords:** tricuspid regurgitation, tricuspid valve disease, transcatheter tricuspid valve interventions, multimodality imaging

## Abstract

Over the last ten years, transcatheter tricuspid valve interventions (TTVIs) have emerged as effective options for symptomatic patients with moderate-to-severe tricuspid regurgitation (TR) who are at prohibitive surgical risk. Successful application of these therapies depends on a patient-tailored, multimodal imaging workflow. Transthoracic and transesophageal echocardiography remain the first-line diagnostic tools, rapidly stratifying TR severity, mechanism, and right ventricular function, and identifying cases requiring further evaluation. Cardiac computed tomography (CT) then provides anatomical detail—quantifying tricuspid annular dimension, leaflet tethering, coronary artery course, and venous access anatomy—to refine candidacy and simulate optimal device sizing and implantation angles. In patients with suboptimal echocardiographic windows or equivocal functional data, cardiovascular magnetic resonance (CMR) offers gold-standard quantification of RV volumes, ejection fraction, regurgitant volume, and tissue characterization to detect fibrosis. Integration of echo-derived parameters, CT anatomical notes, and CMR functional assessment enables the heart team to better select patients, plan procedures, and determine the optimal timing, thereby maximizing procedural success and minimizing complications. This review describes the current strengths, limitations, and future directions of multimodality imaging in comprehensive evaluations of TTVI candidates.

## 1. Introduction

Tricuspid regurgitation (TR) is increasingly recognized as a cause of impaired quality of life and recurrent hospitalizations in the general population [1,2]. Patients with isolated moderate or greater TR face an annual mortality rate of approximately 12.1% [3]. Over the past decade, transcatheter therapies have evolved to offer patients with moderate-to-severe TR options beyond medical management alone [4,5]. Transcatheter therapies have the potential to interrupt the progressive “vicious cycle” of TR by acutely reducing the regurgitant volume, which in turn promotes reverse remodeling of the right atrium and ventricle [6,7,8]. Restoration of effective leaflet coaptation unloads the right heart, attenuates chamber dilatation and wall stress, and correlates with significant improvements in both patient-reported outcomes—such as enhancements in Kansas City Cardiomyopathy Questionnaire (KCCQ) scores and New York Heart Association (NYHA) functional class—and objective functional indices, including 6 min walk distance [6,7]. Early intervention may prevent further deterioration of right ventricular systolic function and avert the onset of fixed pulmonary vascular remodeling and irreversible pulmonary hypertension [9]. Taken together, these data underscore the value of prompt, image-guided transcatheter intervention not only for symptom relief but also to modify disease trajectory and improve long-term cardiopulmonary health. Consequently, they have driven a paradigm shift, reconceptualizing the tricuspid valve from a historically neglected structure to a validated interventional target.

However, without the timely application of integrated multimodality imaging, referrals to heart-valve centers are often delayed, restricting access to percutaneous interventions [10,11]. In particular, the new classification of TR into severe, massive, and torrential allows for more accurate staging of patients, enabling treatment at less advanced stages of the disease [10]. This review examines the complementary roles of echocardiography, cardiac computed tomography (CT), and cardiovascular magnetic resonance (CMR) in enabling early diagnosis and procedural planning. It identifies the current advantages, limitations, and future directions for optimal patient selection to achieve procedural success.

## 2. Role of Echocardiography in Initial Diagnosis

**Figure 1:**
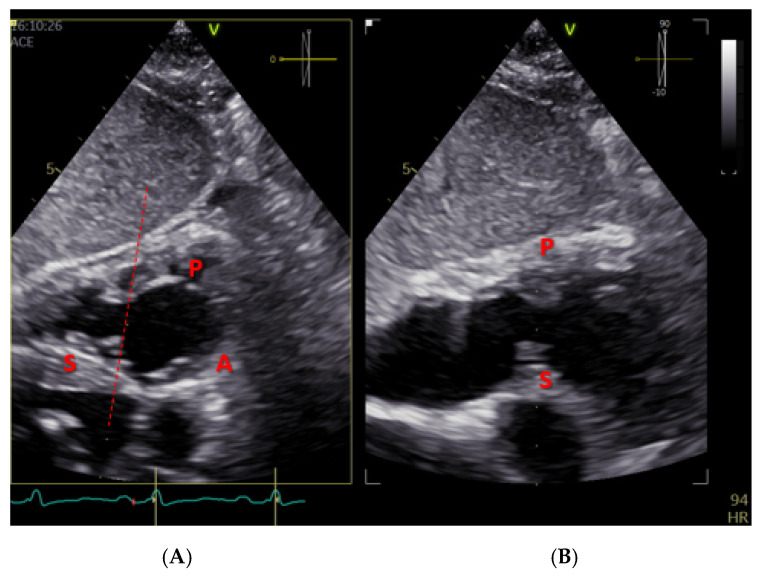
TTE subcostal short-axis view of the tricuspid valve. Panel (**A**) shows a short-axis view of the tricuspid valve using TTE. Panel (**B**) displays the corresponding XPlane image with septal and posterior leaflets. A: anterior leaflet; S: septal leaflet; P: posterior leaflet.

Transthoracic echocardiography (TTE) is the first-line imaging modality for assessing tricuspid regurgitation. It can provide information about tricuspid valve morphology, quantify TR severity, and aid in elucidating the TR mechanism, evaluating right ventricular function, estimating pulmonary artery pressures, and detecting systemic congestion [11]. A comprehensive TTE exam includes multiple views such as parasternal right-ventricular inflow, parasternal short-axis, right ventricle (RV) focus apical four-chamber, and subcostal views [12]. By tilting the probe in the parasternal and subcostal planes, an en face view of all three leaflets can be obtained (Figure 1).

Incomplete imaging of the annulus or suboptimal probe angulation can lead to underestimation or mischaracterization of regurgitant severity, making it essential to pan across the valve to systematically capture its entire circumference [12,13].

**Figure 2:**
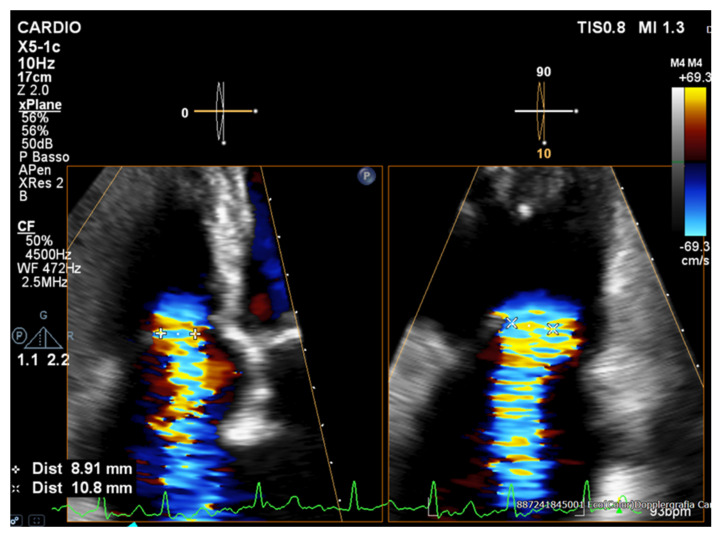
Biplane measurement of the vena contracta for tricuspid regurgitation quantification. The image shows a right ventricular (RV) focus view on transthoracic echocardiography (TTE) with biplane measurement of the vena contracta (VC) for tricuspid regurgitation (TR) quantification.

Current guidelines [10,14] recommend a multiparametric approach—integrating qualitative, semiquantitative, and quantitative measures—to accurately grade tricuspid regurgitation severity. Qualitative assessment should encompass a detailed valve morphology (e.g., leaflet flail or perforation, coaptation gap) and right ventricular remodeling assessment (RV dilation in severe TR), along with visualization of the color-Doppler jet area and continuous-wave (CW) Doppler signal density; a dense, triangular CW profile with early systolic right pressure equalization strongly suggests significant regurgitation. Semiquantitative indices include a dominant PW (Pulsed-Wave) E-wave velocity > 1 m/s, systolic flow reversal in the hepatic veins, and a PISA (proximal isovelocity surface area) radius > 9 mm [10,15]. Biplane measurement of the vena contracta (Figure 2) can further refine the severity grading—a diameter of 7.0–13.9 mm indicates severe TR, 14.0–20.9 mm indicates massive TR, and >21 mm indicates torrential TR—when interpreted alongside quantitative metrics.

Finally, precise quantification using effective regurgitant orifice area (EROA), regurgitant volume, and regurgitant fraction can be used to corroborate semiquantitative findings and ensure robust TR classification [10]. Specifically, in patients with functional tricuspid regurgitation (FTR), the application of correction formulas to PISA measurements is essential for accurate quantification. These corrections account for both the leaflet tethering characteristic of FTR and the relatively low regurgitant flow velocities across the tricuspid valve. The corrected effective regurgitant orifice area (EROAc) can be calculated using the following formula: EROAc = 6.28 × r^2^ × Va × (α/180) × [(Vp − Va)/Vp], where r is the PISA radius, Va is the aliasing velocity, α is the angle between tricuspid leaflets, and Vp is the peak TR velocity [16,17].

This adjustment is necessary because the regurgitant orifice in FTR often forms within a nonplanar annular surface and has an unpredictable, non-circular shape due to asymmetric leaflet tethering. Combined with the lower flow velocities compared to MR, this leads to a flattening of the PISA hemisphere, which can result in significant underestimation of TR severity, affecting up to 20–30% of patients [16,17].

When these correction formulas are applied, significantly higher values for EROA, regurgitant volume (RegVol), and regurgitant fraction (RegFr) are observed—increases of approximately 21 mm^2^, 18 mL, and 24%, respectively—highlighting the importance of incorporating corrected PISA methods in clinical assessments [16,17].

In patients with ventricular FTR (v-FTR), corrected parameters such as EROAc, RegVolc, and RegFrc show a stronger association with clinical outcomes compared to uncorrected values. Conversely, in atrial functional TR (a-FTR), the corrected PISA method did not yield superior prognostic correlation over standard PISA. Importantly, a prognostic threshold for EROAc was identified: values greater than 0.47 cm^2^ were associated with a significantly higher risk of adverse outcomes [16,17].

Accurate mechanism classification is essential, as it directly informs the choice of intervention [10]. In primary TR, intrinsic leaflet pathology—such as prolapse, flail, endocarditis, or rheumatic involvement—predominantly drives regurgitation, and these patients often benefit from techniques aimed at repairing or replacing the diseased leaflets themselves [10]. A-FTR results from isolated tricuspid annular dilation secondary to atrial enlargement (e.g., in atrial fibrillation or heart failure with preserved ejection fraction), with relatively preserved RV geometry. V-FTR arises with RV dilation and dysfunction, due to pulmonary hypertension, left heart disease, or intrinsic RV cardiomyopathy; the leaflets are tethered apically, necessitating strategies that reduce annular dimensions and address leaflet tenting [10]. Finally, Cardiac Implantable Electronic Device (CIED)-related TR stems from permanent pacemaker or defibrillator leads interfering with leaflet motion or causing perforation; secondary mechanisms contributing to CIED-related TR may involve RV desynchronization and adverse remodeling induced by chronic pacing. Management may require lead extraction, repositioning, or targeted percutaneous valve therapies [10]. Tailoring therapy to these distinct pathophysiological substrates maximizes procedural success and long-term valve competence [10].

RV function evaluation is critical for patients undergoing transcatheter tricuspid valve interventions (TTVIs) (Table 1) [10,13,18]. Pre-procedural RV performance holds substantial prognostic value, reflecting the heart’s adaptive reserve following regurgitation reduction. Severely impaired RV function may predict limited benefit or higher procedural risk, underscoring the importance of tailored patient selection [6,7,10,19].

Notably, a mild decline in TTE RV functional parameters—such as tricuspid annular plane systolic excursion (TAPSE) and fractional area change (FAC)—has been frequently observed within 30 days after a TTVI [10]. This early decrease, which does not negatively influence the prognosis, is likely attributable to the unmasking of latent RV dysfunction that was previously obscured by the reduced afterload state inherent to severe tricuspid regurgitation [10,17]. Supporting this interpretation, studies have consistently demonstrated an increase in forward stroke volume after a TTVI, suggesting improved RV efficiency despite apparent declines in contractile indices [19,21].

Recent data suggest that the prognostic role of RV function in this setting is more nuanced than previously thought. Schlotter et al. described a U-shaped relationship between baseline RV function and clinical benefit from TTVI, highlighting that patients with mid-range RV function (e.g., TAPSE 13–17 mm) derive the most significant survival advantage from intervention compared to conservative management. In this group, TTVIs were associated with improved one-year outcomes comparable to those with preserved RV function, whereas patients with severely reduced RV function (TAPSE < 13 mm) experienced no clear survival advantage, potentially due to advanced, less reversible remodeling. Conversely, those with preserved RV function may appear too early in the disease course to demonstrate survival benefits within the limited follow-up timeframe [21].

Thus, optimal timing is crucial: patients in the early stages of RV dysfunction may exhibit a greater potential for reverse remodeling and symptomatic improvement following a TTVI. Accordingly, integrating RV function into clinical decision-making—both pre- and post-procedure—can optimize the outcomes and guide the allocation of advanced therapies more effectively.

Pulmonary hypertension (PH) represents another major determinant of prognosis following a TTVI [10]. TTE estimation of systolic pulmonary artery pressure (sPAP) often underestimates PH, particularly in the context of large coaptation gaps and severe TR, due to early systolic pressure equalization [22]. Recent evidence [23] highlighted that elevated invasive sPAP is significantly associated with worse outcomes following T-TEER. In a large multicenter cohort, an sPAP above 46 mmHg—observed in nearly 43% of the patients—was linked to a marked reduction in two-year survival free from heart failure hospitalization (HFH) [23]. Invasive diastolic PAP (dPAP) also emerged as an independent prognostic factor, with both parameters demonstrating comparable predictive values. Moreover, insights from the EuroTR registry, which uniquely includes patients with both post- and precapillary PH who are often excluded from clinical trials—further emphasized the prognostic relevance of PH burden. Importantly, no significant differences in HFH-free survival were observed between these PH subtypes, reinforcing the need for individualized hemodynamic assessment rather than exclusion based only on PH classification. These findings support the routine use of right heart catheterization as an integral part of the diagnostic workup [23].

To overcome the limitations of relying solely on TAPSE and sPAP in the assessment of patients being considered for a TTVI, further approaches such as RV–pulmonary artery (PA) coupling and artificial intelligence-based prediction models are gaining increasing relevance. In certain patients with severely impaired RV function, the abrupt correction of severe TR may precipitate acute RV failure due to a sudden rise in afterload. As such, the ratio of TAPSE to sPAP—serving as a surrogate marker of RV–PA coupling—has emerged as a powerful prognostic indicator across various structural heart interventions [18,22]. A lower baseline TAPSE/sPAP ratio (<0.4) has been associated with a greater burden of comorbidities—such as diabetes, prior myocardial infarction, and renal dysfunction—suggesting a more advanced or chronically maladapted disease state [18]. These patients may present later in the disease course, when irreversible structural and functional deterioration of the RV has already occurred [18]. However, the accuracy of echocardiographic sPAP in severe TR is often compromised by rapid pressure equalization across the regurgitant orifice, leading to systematic underestimation of pulmonary pressures [18].

Advanced machine learning techniques have been proposed to improve risk stratification and overcome these echocardiographic limitations [24,25]. Specifically, an extreme gradient boosting (XGB) algorithm was developed to estimate mean pulmonary artery pressure from non-invasive echocardiographic data. It offers a promising alternative to right heart catheterization. The key variables included in the model were left ventricle ejection fraction (LVEF), left atrium area, left ventricle end-systolic diameter, basal RV diameter, TAPSE, TR vena contracta width, right atrium area, inferior vena cava (IVC) diameter, sPAP, and tricuspid valve EROA [24,25].

This approach reaffirms the role of PH in post-TTVI mortality and advances precision medicine in structural heart interventions [24,25].

## 3. Multimodality Imaging for Procedural Planning

**Figure 3:**
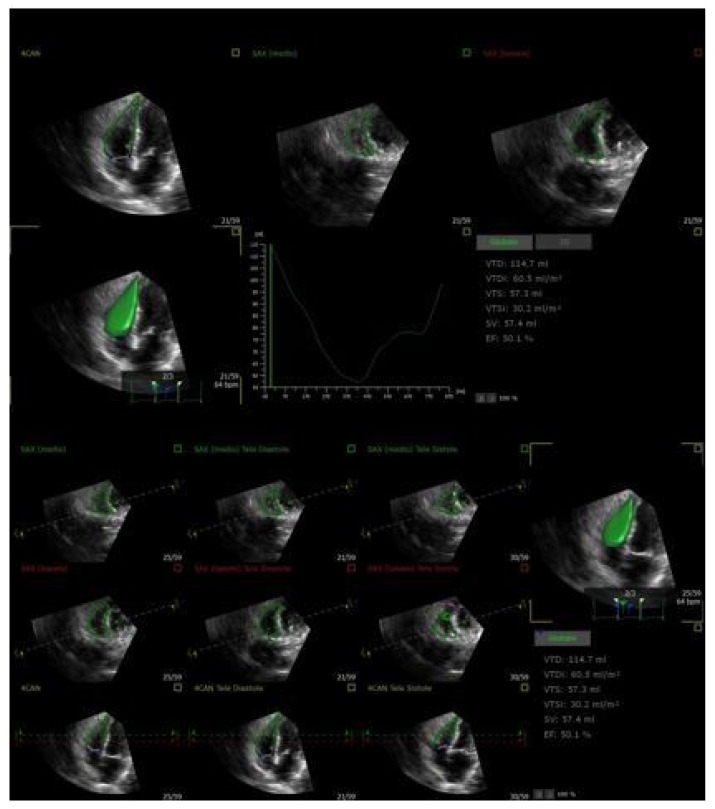
Three-dimensional assessment of RV volume and function. This figure illustrates the use of 3D echocardiography to evaluate right ventricular (RV) morphology and performance. The RV end-diastolic and end-systolic volumes are calculated from full-volume datasets, allowing for the accurate estimation of the RV ejection fraction (RVEF). Compared to 2D measurements, 3D imaging provides a more reliable quantification. This technique is particularly useful in patients with tricuspid regurgitation, where RV size and function are crucial determinants of prognosis and therapeutic decision-making.

Considering the numerous benefits over two-dimensional imaging, three-dimensional transthoracic echocardiography (3D TTE) is essential to the functional evaluation of the RV (Figure 3) [26]. Specifically, 3D TTE overcomes the foreshortening restrictions of 2D techniques by enabling volumetric assessment of the RV dimensions and ejection fraction (RVEF). It makes it possible to precisely see the entire RV, including the apex, outflow tract, and inflow [10,26].

**Figure 4:**
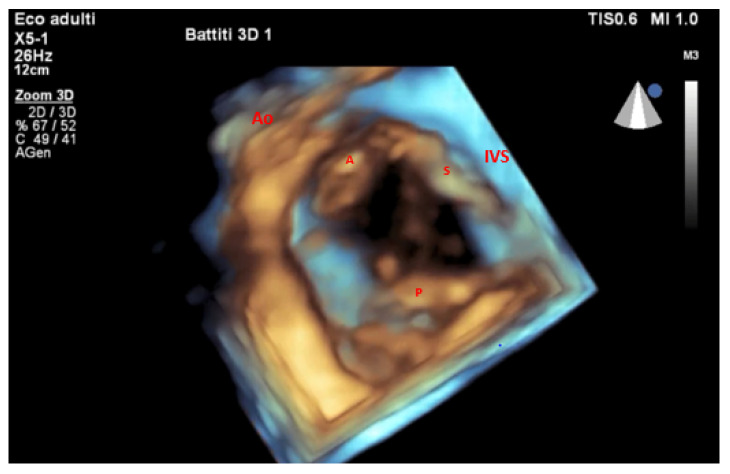
TTE 3D view of the TV. Three-dimensional TTE ventricular view clearly showing the tricuspid valve leaflets. S: septal; A: anterior; P: posterior; Ao: aortic valve; IVS: interventricular septum; TV: tricuspid valve.

Consequently, when compared to traditional 2D techniques, 3D-derived measurements of RV size and function reveal better concordance with CMR data [20]. The efficiency and reliability of 3D RV volumetric analysis have been further improved by advances in automated software, which also make it easier to rapidly obtain clinically relevant parameters [26]. Evaluating RV function is essential in predicting outcomes after TTVI. Accurate evaluation using 3D imaging, including automated evaluation of RVEF and RV–pulmonary arterial coupling via the RVEF/PASP ratio, enhances clinical decision-making and risk stratification [27,28]. Moreover, 3D transesophageal echocardiography (3D TEE) offers superior anatomical resolution for a detailed evaluation of tricuspid leaflet morphology, annular geometry, and regurgitation mechanisms, while 3D TTE offers a strong noninvasive modality for an exhaustive assessment of right ventricular size, shape, and systolic performance. As a result, TEE is exceptionally useful for intraprocedural guiding and pre-procedural strategy. Therefore, a multimodal imaging strategy that guarantees both anatomical and functional precision in patient selection, procedure design, and post-interventional follow-up requires using both 3D TTE (Figure 4) and 3D TEE.

Advanced techniques such as biplane and three-dimensional echocardiography—especially when combined with multiplanar reconstruction (MPR)—have become indispensable tools because of the tricuspid annulus (TA)’s complex, three-dimensional shape, which can be difficult to fully appreciate with standard two-dimensional imaging. These techniques enable clinicians to measure the annulus more precisely, allowing for a better understanding of the underlying causes of regurgitation and for clearly identifying the origin of the regurgitant jet [29]. TEE biplane imaging, with and without color Doppler, serves as a valuable initial step in delineating valve morphology and identifying the mechanism and severity of regurgitation [29].

Compared to conventional 2D imaging, biplane acquisition allows for direct visualization of commissures, thereby facilitating leaflet identification and enhancing anatomical classification. This approach enables a more intuitive alignment with the Hahn classification of tricuspid valve morphology, which recognizes that the classic three-leaflet configuration (Type I: anterior, septal, and posterior leaflets) only occurs in 28% to 58% of cases. In many patients, the tricuspid valve exhibits accessory leaflets that deviate from the canonical tri-leaflet model, complicating the interpretation using 2D imaging alone [30].

Mid and deep-esophageal RV inflow–outflow views (typically 60–100°) can be used to assess the leaflet length, coaptation gap, tenting area, and presence of flail segments. Complementary transgastric short-axis views (20–60°) provide simultaneous visualization of all three valve leaflets, enabling identification of complex morphologies and the extent of the regurgitant jet [31].

Importantly, the trileaflet configuration of the TV often results in an irregular, non-circular regurgitant orifice—violating the key assumptions of the proximal isovelocity surface area (PISA) method. As such, determining the 3D planimetry of the vena contracta (VC) using MPR and color Doppler offers a more accurate assessment of regurgitation severity [30]. By aligning 3D datasets along orthogonal planes (coronal, sagittal, and transverse), the regurgitant vena contracta area (VCA) can be measured in cross-section, typically during end-systole [32]. According to the Tricuspid Valve Academic Research Consortium, the 3D VCA thresholds for severe, massive, and torrential TR are 75–94.9 mm^2^, 95–114.9 mm^2^, and ≥115 mm^2^, respectively [10].

**Figure 5:**
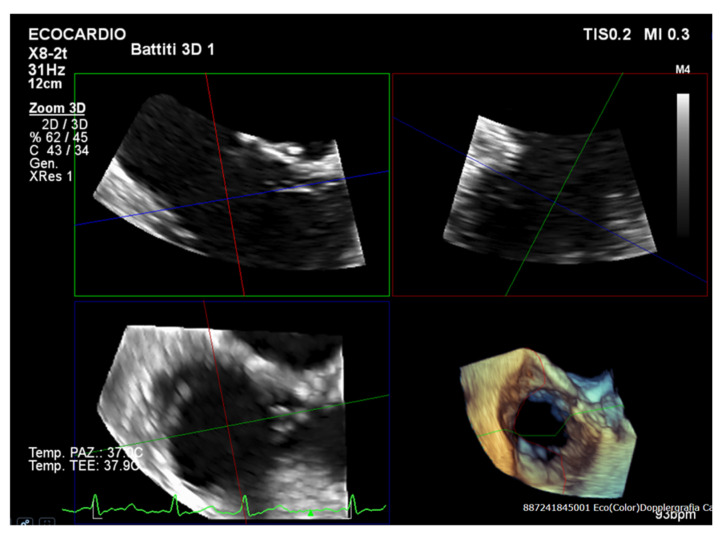
Multiplanar reconstruction of the tricuspid valve annulus. Measurement of tricuspid annular dimensions using TEE-derived MPR imaging. The green box shows the RV inflow–outflow view, the red box shows the septum/lateral view of the tricuspid annulus, and the blue box displays the cross-sectional en face view of the annulus.

MPR also enables precise quantification of the tricuspid annular dimensions (Figure 5), including the annular area and perimeter in the short-axis plane, and the maximum septolateral and anteroposterior diameters in the coronal and sagittal orientations, respectively [33,34].

**Figure 6:**
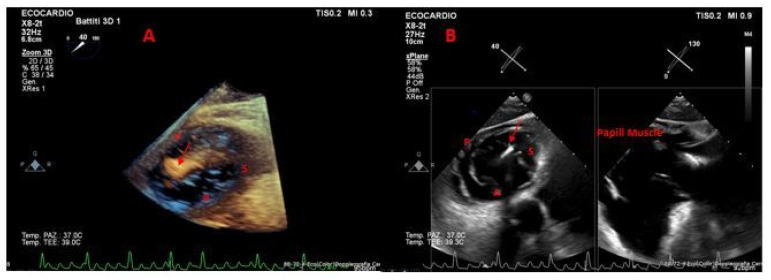
Lead positioned at the posteroseptal commissure of the tricuspid valve. Panel (**A**): TEE 3D image of the TV with the lead indicated by the arrow. Panel (**B**): Transgastric view with X-plane imaging showing the lead in the posteroseptal commissure, as evidenced by the underlying papillary muscle. S: septal; A: anterior; P: posterior; Papill Muscle: papillary muscle.

Moreover, 3D TEE is instrumental in identifying CIED-related TR by determining the relationship between pacing leads and valve anatomy (Figure 6) and guiding decisions on potential extraction or procedural planning to mitigate interference [35].

Annuloplasty planning also benefits from 3D annular measurements, including the distance between anteroseptal and posteroseptal commissures, which are used to size the ring appropriately [36]. In addition to MPR, several semi-automated software platforms have been developed to quantify tricuspid annular dimensions in TTVI planning [37,38]. These tools demonstrate excellent agreement with MPR-derived measurements, streamlining annular sizing through automated landmark detection and contouring [37,38]. However, their use remains limited to a small number of specialized centers, highlighting the need for broader dissemination and validation to enhance procedural standardization [37,38].

Together, 3D TEE and 3D TTE offer a synergistic approach that captures the full spectrum of anatomical complexity and hemodynamic consequences in patients undergoing transcatheter tricuspid valve therapies.

However, the anterior position of the tricuspid valve relative to the chest wall often limits optimal visualization with two-dimensional echocardiography, consequently restricting the quality of 3D imaging as well [39]. To overcome these limitations, cardiac CT offers superior spatial and endocardial resolution of right-sided heart structures [40]. Moreover, the isotropic resolution of CT allows for the reconstruction of datasets in any desired imaging plane, providing a comprehensive and anatomically accurate assessment [40,41]. Considering these complex valve and annular geometries—and given that meticulous pre-procedural planning is the cornerstone of procedural success—CT occupies a central role in the multimodality imaging workflow for tricuspid regurgitation [41].

Cardiac CT enables precise quantification of the TA by reconstructing orthogonal planes from standard right-ventricular two- and four-chamber views at end-diastole and mid-systole [42]. From these reconstructions, a true short-axis cross-section of the annulus can be generated, resulting in planar measurements that have been linked to TR severity—mean perimeters of 148 ± 16 mm and areas of 1612 ± 295 mm^2^ differentiate patients with severe or worse (grade ≥ 3) regurgitation [43]. However, because the TA is a non-planar structure, simple 2D measurements frequently underestimate its true dimensions.

Semi-automated 3D post-processing platforms such as 3mensio Structural Heart have been introduced to overcome these limitations [42,44]. By importing isotropic CT datasets, these tools automatically identify key annular landmarks in three dimensions and generate interactive multiplanar reconstructions alongside fully rendered 3D models. Operators can refine the annular contour throughout the cardiac cycle, obtaining accurate metrics—annular area, perimeter, and septolateral and anteroposterior diameters—while preserving the valve’s complex geometry [44].

As Praz et al. have suggested, 3D-derived annular measurements are particularly well suited to annuloplasty planning, since repair devices must conform to the valve’s undulating, non-planar surface. In contrast, 2D-planar sizing—based on conventional short-axis CT reconstructions—may be sufficient when sizing a transcatheter prosthesis for valve replacement, where planar dimensions drive device selection [45].

**Figure 7:**
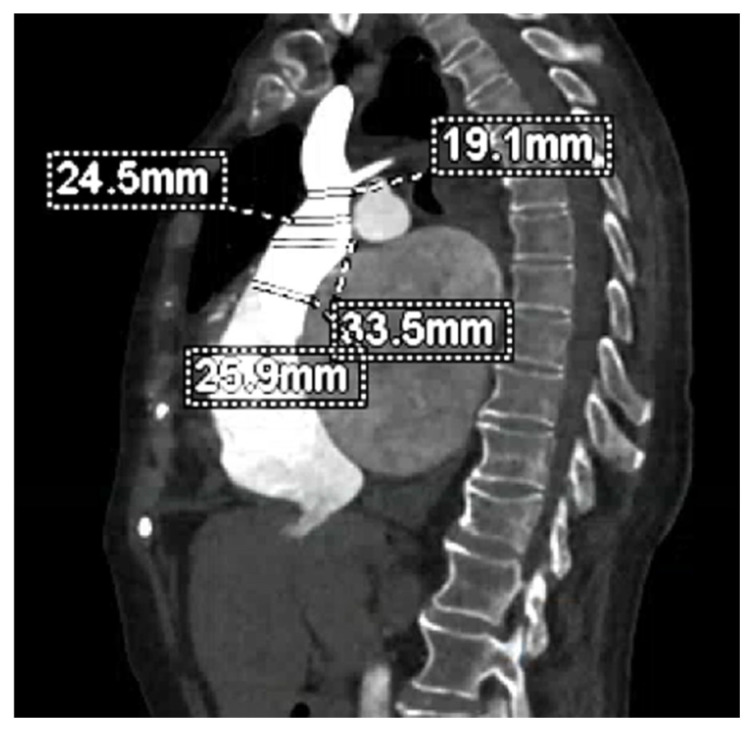
CT-based sizing of the superior vena cava. Cardiac CT image showing superior vena cava sizing in the context of pre-procedural planning for heterotopic caval valve implantation in a patient with torrential tricuspid regurgitation.

While cardiac CT is not routinely incorporated into pre-procedural planning for transcatheter tricuspid edge-to-edge repair (T-TEER), it has a pivotal role in annuloplasty strategies [41]. The right coronary artery (RCA) courses close to the TA and is at risk for compression, spasm, or injury during ring implantation; precise CT-based delineation of its anatomical trajectory is therefore essential to minimize coronary complications. Furthermore, CT is invaluable when planning heterotopic valve implantation in the IVC (Figure 7) [41].

By acquiring mid-systolic axial datasets, CT enables accurate measurement of IVC dimensions just below the right atrium and at the ostium of the first hepatic vein—critical parameters for selecting the appropriate device size. Although right ventricular outflow tract obstruction is less common than left ventricular outflow tract compromise in transcatheter mitral valve implantation, pre-procedural CT planning for orthotopic tricuspid valve replacement is nevertheless indispensable. Cardiac CT provides accurate annular sizing and enables virtual simulation of the prosthesis landing zone, including assessment of the distance between the device and the RV apex [41]. Additionally, CT planning assists in predicting the optimal angle projection during valve implantation. Further, CT reconstructions make it possible to see the moderator band and right ventricular trabeculations in great detail, which aids in anticipating and preventing possible mechanical interference. CT minimizes the risk of complications by ensuring appropriate device selection, positioning, and deployment through the integration of these data and simulations of the procedural process [41]. However, CT-derived RV volumes and ejection fractions frequently exceed the real values [46].

CMR remains the gold-standard modality for accurate assessment of RV volumes and function [15,46].

However, current guidelines only recommend using CMR in this scenario for patients with suboptimal echocardiographic acoustic windows where tricuspid regurgitation severity remains uncertain [15].

**Figure 8:**
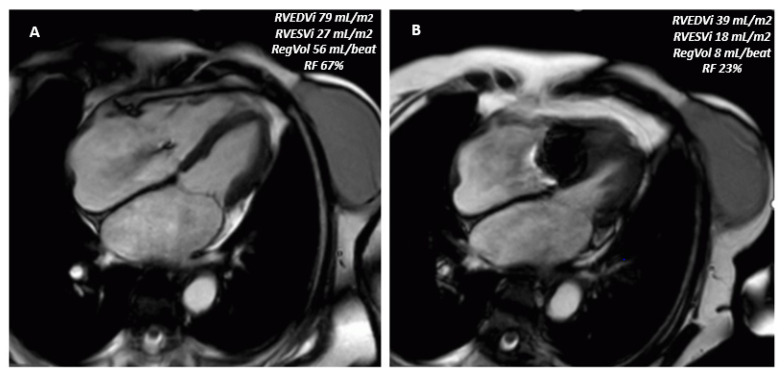
Pre- and post-T-TEER CMR. Panel (**A**): Pre-T-TEER cine SSFP sequences. Panel (**B**): Post-T-TEER cine SSFP sequences showing reverse remodeling of the right ventricle. Phase contrast sequences enable quantification of regurgitant volume reductions, calculated as the difference between left ventricular stroke volume and pulmonary forward flow. T-TEER: transcatheter tricuspid edge-to-edge repair; SSFP: Steady State Free Procession; RVEDVi: Right Ventricle End-Diastolic Volume Index; RVESVi: Right Ventricle End-Systolic Volume Index; Reg Vol: regurgitant volume; RF: regurgitant fraction.

Unlike CT, CMR involves no ionizing radiation or iodinated contrast agents, rendering it safe for patients with chronic kidney disease. Steady-state free precession cine sequences afford highly accurate measurements of the right and left ventricular volumes, ejection fraction, and forward stroke volume, while phase-contrast flow imaging permits direct quantification of the tricuspid regurgitant volume and fraction (Figure 8) [46].

Notably, CMR and 3D echocardiography show excellent agreement in TR quantification. Doldi et al., who proposed a five-class CMR-based grading system for TR severity, demonstrated that CMR-derived regurgitant volumes correlate closely with 3D echo measurements, underscoring the complementary value of these modalities in a multimodal imaging strategy [47]. CMR 4D flow imaging now enables direct quantification of tricuspid regurgitant volume [48].

**Figure 9:**
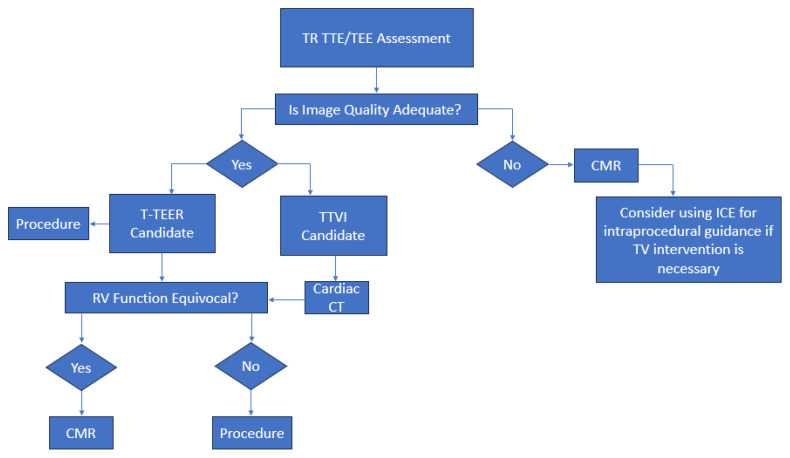
Multimodal imaging workflow for patients with significant tricuspid regurgitation referred for percutaneous therapy. The figure illustrates the workflow for evaluating patients with significant tricuspid regurgitation who are candidates for percutaneous therapy. All patients initially undergo TTE and TEE evaluations to assess the TR severity, mechanism, and RV function. In cases of inadequate acoustic windows or inconclusive echo findings, CMR is performed for definitive evaluation. Patients with adequate echocardiographic imaging who meet the criteria for T-TEER proceed directly to that intervention. Those with other TTVIs planned undergo cardiac CT to confirm anatomic feasibility, including annular sizing and implantation planning. Finally, any patient whose RV function remains uncertain should undergo CMR prior to the procedure to guide heart-team decision-making. TR: tricuspid regurgitation; TTE: transthoracic echocardiography; TEE: transesophageal echocardiography; RV: right ventricle; T-TEER: tricuspid transcatheter edge-to-edge repair; TTVI: transcatheter tricuspid valve interventions; CMR: cardiovascular magnetic resonance; CT: computed tomography.

Beyond functional analysis, late gadolinium enhancement reveals focal myocardial fibrosis, and native T1 mapping or extracellular volume quantification assesses diffuse interstitial remodeling. However, major artifacts impairing image quality restrict the use of CMR. These might be caused by intracardiac devices, such as pacemaker or defibrillator leads, and arrhythmias. Long scan times and breath holding may not be tolerated, especially by low-compliance patients. Furthermore, only specialized centers may be able to use CMR scanners due to their relative scarcity and high cost [46]. A truly patient-tailored approach to TTVI hinges on the seamless integration of complementary imaging modalities (Figure 9).

TTE and TEE serve as the initial gatekeepers, rapidly stratifying patients by TR severity, mechanism, and RV function, and flagging those who require more advanced assessments. Cardiac CT then provides high-resolution anatomical details—quantifying the annular dimensions, leaflet tethering, coronary course, and IVC size—to refine candidacy, select the device type (repair versus replacement), and simulate the implantation angles. In cases where echocardiographic windows are poor or functional measurements remain equivocal, CMR offers gold-standard quantification of the RV volume, ejection fraction, and regurgitant flow, and tissue characterization to exclude significant fibrosis that may portend poor remodeling potential. By synthesizing the echo-derived hemodynamics and TR mechanism, CT-based anatomical landmarks, and CMR-quantified RV function and tissue characteristics, the heart team can identify the patients most likely to benefit from intervention, optimize the procedural strategy, and minimize the risk of futility or complications. This multimodality imaging workflow thus ensures that each patient’s unique anatomy and physiology guide the choice, timing, and technique of tricuspid valve therapy.

## 4. Imaging-Based Eligibility Criteria

Multimodality imaging is indispensable in the work-up of patients referred for TTVIs, as it identifies predictors of procedural failure and informs patient-tailored therapy (Table 2) [36,49]. Candidates with severe or worse TR should be excluded if they exhibit advanced RV systolic dysfunction or fixed pulmonary hypertension, both of which portend poor outcomes [6,7]. A left ventricular ejection fraction of ≥30% is generally required [36].

Further, patients with significant aortic, mitral, or pulmonary valve disease demanding priority intervention must be ruled out [48]. Finally, optimal procedural guidance hinges on adequate echocardiographic windows, specifically mid- and deep-esophageal TEE as well as transgastric views, to visualize valve anatomy and device positioning throughout the intervention [50].

Furthermore, intervention-specific feasibility must be rigorously assessed. In candidates for T-TEER, a rheumatic etiology of regurgitation must be excluded, as must any coexistent tricuspid stenosis that could be exacerbated by leaflet approximation [49]. Moreover, when tricuspid regurgitation is driven by CIED lead interference with leaflet coaptation as in lead-associated tricuspid regurgitation, type A (LTR-A), percutaneous edge-to-edge repair is not appropriate; instead, management should prioritize lead revision or extraction and alternative valve therapies [49].

The optimal echocardiographic criteria for T-TEER candidacy hinge on leaflet anatomy and jet location. Specifically, the coaptation depth should not exceed 10 mm, and the coaptation gap must be <8.5 mm—ideally <4 mm—to ensure secure device attachment [36,49,51,52]. The leaflet lengths must exceed 10 mm to provide adequate tissue for clip capture [36,51,52]. Finally, the regurgitant jet should arise centrally or at the antero-septal commissure, facilitating precise device alignment along the line of coaptation. These thresholds maximize the probability of effective leaflet approximation and durable TR reduction [36,50].

In patients who are not candidates for edge-to-edge repair due to advanced disease features, evaluation for transcatheter tricuspid valve replacement (TTVR) is indicated [49]. Regarding T-TEER candidacy, the most frequent screening failure arises from a coaptation gap exceeding 8.5 mm. In contrast, candidates for TTVR are most often excluded because of an excessively large TA [49]. In Table 3, the main differences in patient selection criteria for T-TEER and TTVR are summarized. Although next-generation device sizes are bigger, an annular perimeter > 160 mm at end-diastole or a 2D annular diameter > 60 mm remains highly predictive of exclusion with the current systems [49]. In particular, the TRISCEND trial represents a pivotal study evaluating the safety and efficacy of TTVR systems (EVOQUE) in patients with moderate or worse symptomatic TR. Eligible patients were those without anatomical limitations to device implantation, without severe pulmonary hypertension (PAPs < 70 mmHg), and without severe right or left ventricular dysfunction (left ventricular EF > 25%). Additionally, patients with a pacemaker lead implanted within the previous three months and those with severe renal failure (eGFR had to be >25 mL/min/1.73 m^2^) were excluded [7].

The key differences in patient selection criteria between transcatheter tricuspid valve repair (T-TEER) and transcatheter tricuspid valve replacement (TTVR). The table outlines the factors that influence the choice of approach based on current evidence and ongoing clinical trials. ICE: intracardiac echocardiography.

When a non-femoral approach is required, additional anatomic constraints come into play: a right atrial length exceeding 6–7 cm may complicate transatrial or transjugular delivery, and a right internal jugular to superior vena cava distance < 14 mm can impede sheath advancement [36]. Other considerations include the course of the right coronary artery, the risk, albeit rare, of right ventricular outflow tract obstruction, and confirmation of a favorable coaxial deployment angle to ensure stable device seating [36]. Beyond anatomical criteria, a comprehensive pre-procedural assessment must also include evaluation of the patient’s hemorrhagic risk and the feasibility of long-term anticoagulation, which is essential after tricuspid valve replacement to prevent device thrombosis [7].

Dedicated coaptation spacer devices may be employed to enhance leaflet coaptation. Thus, echocardiographic screening must confirm a coaptation depth of less than 10 mm and a coaptation gap of less than 18 mm to ensure that the spacer can effectively bridge the regurgitant orifice [36]. Pre-procedural CT is then used to verify that the left subclavian or axillary venous access route will accommodate the device delivery system—requiring a vessel diameter exceeding 7.1 mm for a 12 mm spacer or 8.3 mm for 15–18 mm spacers—and to measure the key intracardiac dimensions [36]. Specifically, the distance from the relevant papillary muscle to the tricuspid annular plane must exceed 20 mm, and the distance from the papillary muscle to the septum must be at least 15 mm for a 15 mm device or 18 mm for an 18 mm device [36]. These spatial relationships ensure that the spacer can be positioned without impinging on adjacent structures and will remain securely in place during the cardiac cycle [36].

Alternatively, CT-derived assessment of annular geometry is paramount for patients suited to restrictive annuloplasty techniques (such as the Trialign system) [36]. Candidates must have a tricuspid annular diameter under 55 mm and a posterior annular depth of 2–4 mm to allow for deployment of the pledget-based plication mechanism [36]. In addition, the course and proximity of the right coronary artery relative to the annulus must be carefully delineated to avoid coronary compression or injury during annular cinching [36].

For patients deemed ineligible for both transcatheter repair and orthotopic valve replacement, heterotopic caval valve implantation offers a palliative alternative to alleviate venous congestion [10]. In this scenario, pre-procedural CT planning is crucial for defining the appropriate landing zones within the IVC. Specifically, the IVC diameter measured at end-systole just below the right atrial junction and at the ostium of the first hepatic vein must fall below device-specific thresholds—typically <35 mm for the TricValve system and <42 mm for the Tricento prosthesis—to ensure secure anchoring and minimize the risk of migration [36]. Therefore, precise CT reconstructions in multiple planes are needed to guide device selection and positioning in this heterotopic treatment strategy [36].

Recently, a five-point echocardiographic scoring system, the GLIDE score, was proposed to predict procedural success in patients referred for T-TEER [50]. In this imaging-based scoring system, one point is assigned for each of the following adverse features: a coaptation gap of 6 mm or greater; a regurgitant jet located in the posteroseptal, anteroposterior, or diffuse region; a star shape for the jet origin on color Doppler; suboptimal image quality on intraprocedural TEE; and a high density of subvalvular chordae [50]. Clinical validation has shown that patients with total GLIDE scores of 0 or 1 achieve procedural success in 97% of cases, while 61% of those with scores of 2 or 3 have successful procedures and only 14% of those scoring 4 or 5 have successful procedures [50].

In addition to echocardiographic metrics, CT-based criteria have also been explored to predict procedural success in T-TEER, even though CT is not routinely performed before this intervention [53]. Among the anatomic measures evaluated, RV length emerged as the only independent CT predictor of procedural success [53]. Specifically, a longer RV length and a shorter distance from the IVC to the TA were each associated with prolonged fluoroscopy time and greater overall procedural complexity [53].

## 5. Challenges and Future Perspectives

A patient-specific, imaging-guided strategy remains the cornerstone of procedural success in transcatheter tricuspid intervention.

Looking ahead, 3D-printed heart models offer a pathway to even more individualized planning. By converting high-resolution CT or CMR datasets into tactile, life-size replicas, soft-material 3D prints recapitulate the true surgical anatomy far more intuitively than on-screen reconstructions alone [54]. Using these physical models, the heart team can predict anatomical challenges, improve device sizing and trajectory, and practice device motions ex vivo. Thus, 3D printers offer learners a priceless simulation platform that reduces patient risk during the learning curve, and increases hands-on familiarity and procedural confidence [54]. Despite the obvious pros, the current long lead times for modeling and printing and high manufacturing costs prevent broad implementation [54]. Pre-procedural planning could be transformed into a customized process as 3D printing technology develops and becomes more affordable, leading to further improvements in long-term results, safety, and efficiency [54]. Furthermore, in cases of inadequate pre-procedural imaging in candidates for TTVIs, intracardiac echocardiography (ICE) plays a fundamental role in procedural guidance. It allows for precise device orientation and leaflet capture without shadowing, particularly in patients with poor acoustic windows and/or contraindications to TEE. ICE provides high-resolution, real-time imaging from within the heart, enabling the optimal visualization of valve anatomy and device–tissue interactions. This is especially valuable in complex anatomies where traditional imaging modalities fall short. Despite its advantages, its widespread use is limited by cost considerations, operator experience, and availability across different centers. Nonetheless, as technology advances and experience grows, ICE is expected to become increasingly integrated into structural heart interventions [55].

## 6. Conclusions

The evolution of transcatheter therapies is transforming the management of tricuspid regurgitation, from medical treatment to percutaneous intervention. Central to this paradigm shift is the integration of multimodality imaging, including echocardiography, cardiac CT, and CMR [46]. By utilizing the complementary strengths of these modalities, the heart team can tailor interventions to each patient’s unique anatomy. Ultimately, applying an imaging-centric workflow is crucial for enhancing procedural safety, optimizing efficacy, and ensuring durable results in transcatheter tricuspid valve therapy. However, it is important to note that not all imaging modalities are necessary or appropriate in every case. Imaging should be selected based on clinical indication, image quality, patient characteristics, and local resource availability. A flexible and patient-centered approach allows for effective planning and guidance while avoiding unnecessary testing or excessive resource use. This ensures a pragmatic balance between optimal imaging and procedural efficiency in real-world settings.

## Figures and Tables

**Table 1:** Multimodal assessment of right ventricular function before and after transcatheter tricuspid valve intervention [10,20].

Method	Parameter(s)	Advantages	Limitations
**2D Echocardiography**	TAPSE (tricuspid annular plane systolic excursion)	Simple, widely available, reproducible	Can be pseudo-normal in severe TR
	S′ (tissue Doppler systolic velocity)	Easy, less load-dependent than TAPSE	Influenced by angle and operator skill
	FAC (fractional area change)	Evaluates global RV function	Suboptimal image quality may limit accuracy; in a severe TR setting, it cannot distinguish between anterograde and retrograde flow.
Strain Imaging (Speckle Tracking)	RV longitudinal strain	Early marker of dysfunction; higher prognostic significance in contrast to traditional metrics	Limited standardization, inter-vendor variability
**3D Echocardiography**	RV ejection fraction (RVEF), RV volumes	Volumetric, less geometrically biased	Requires good acoustic window, technically demanding, poorly characterized prognostic impact in the context of TTVIs
**Cardiac CMR**	RVEF, RV volumes, fibrosis detection	Gold standard for RV volumes and function; not operator-dependent	Limited availability, contraindications (e.g., devices), expensive, does not consider the direction of the flow
**Cardiac CT**	RV volumes (limited functional info)	High spatial resolution; useful for anatomy and procedural planning	Not ideal for function, radiation exposure, no direct contractility assessment
**Hemodynamic Assessment**	Right atrial pressure, pulmonary pressures	Direct measurement; useful during the procedure	Invasive
**Pulmonary Artery Coupling**	TAPSE/PASP ratio	Reflects RV–arterial interaction; prognostic value	Requires accurate PASP estimation

This table summarizes the key imaging and hemodynamic modalities used to assess right ventricular (RV) function in the context of transcatheter tricuspid valve interventions (TTVIs). Each technique offers unique insights into RV performance, structure, and adaptation, both pre- and post-procedure. The advantages and limitations of each method are discussed to guide optimal clinical application.

**Table 2:** Imaging considerations for patient selection for different TR treatment strategies [36,49].

Treatment Strategy	Imaging Considerations for Patient Selection
T-TEER	Coaptation gap < 8.5 mm; Not suitable in case of TV stenosis or LTR-A.
Annuloplasty	Tricuspid annulus diameter < 55 mm; Not suitable in case of severe tethering, RCA proximity, or LTR-A.
Coaptation Device	Coaptation gap < 18 mm; Ensure adequate venous access and sufficient distance between papillary muscles, tricuspid annulus, and septum to allow for safe and effective device delivery and deployment
TTVR	Not suitable in case of severe RV dysfunction, extreme annular dilation, or unfavorable device access angle.
Heterotopic Valve Implantation	Requires adequate caval diameters and intercaval distance; Contraindicated if right atrium to hepatic veins distance is <10–12 mm.

**Table 3:** Differences in patient selection criteria: T-TEER vs. TTVR candidates [6,7].

Criteria	T-TEER	TTVR
TR Etiology	Preferably functional TR	Both functional, PMK-associated and primary TR
TR Jet	Preferably central TR jet origin	Effective regardless of TR jet origin
Anatomical Suitability	Requires adequate leaflet tissue for grasping or coaptation	Less dependent on native leaflet morphology
Coaptation Gap	<8.5 mm generally preferred	Larger coaptation gaps acceptable
Pacemaker/ICD Leads	Not effective if TR is lead-induced	Challenging if patient recently received lead implantation (within 3 months)
Durability Considerations	Long-term durability uncertain	Device durability under investigation
Imaging Requirements	High-quality TEE or ICE for guidance	Requires comprehensive multimodality imaging (TEE and/or ICE, and CT)

## Abbreviations

The following abbreviations are used in this manuscript:

TTVI	Transcatheter Tricuspid Valve Intervention
TRJ	Tricuspid Regurgitation
CT	Computed Tomography
CMR	Cardiovascular Magnetic Resonance
KCCQ	Kansas City Cardiomyopathy Questionnaire
NHYA	New York Heart Association
TTE	Transthoracic Echocardiography
RV	Right Ventricle
CW	Continuous-Wave
PW	Pulsed-Wave
EROA	Effective Regurgitant Orifice Area
aFTR	Atrial Functional TR
vFTR	Ventricular Functional TR
CIED	Cardiac Implantable Electronic Device
FAC	Fractional Area Change
EF	Ejection Fraction
TAPSE	Tricuspid Annular Plane Systolic Excursion
PH	Pulmonary Hypertension
sPAP	Systolic Pulmonary Artery Pressure
HFH	Heart Failure Hospitalization
PA	Pulmonary Artery
XGB	Extreme Gradient Boosting
IVC	Inferior Vena Cava
3D	Three-Dimensional
MPR	Multiplanar Reconstruction
TA	Tricuspid Annulus
Reg Vol	Regurgitant Volume
VC	Vena Contracta
VCA	Vena Contracta Area
T-TEER	Transcatheter Tricuspid Edge-to-Edge Repair
RCA	Right Coronary Artery
TEE	Trans Esophageal Echocardiography
LTR-A	Lead-Associated Tricuspid Regurgitation Type A
TTVR	Transcatheter Tricuspid Valve Replacement
